# A thematic study: impact of COVID-19 pandemic on rare disease organisations and patients across ten jurisdictions in the Asia Pacific region

**DOI:** 10.1186/s13023-021-01766-9

**Published:** 2021-03-05

**Authors:** Claudia Ching Yan Chung, Yvette Nga Chung Ng, Ritu Jain, Brian Hon Yin Chung

**Affiliations:** 1 Department of Paediatrics and Adolescent Medicine, The University of Hong Kong, Hong Kong, Hong Kong SAR; 2 Asia Pacific Alliance of Rare Disease Organisations, Singapore, Singapore; 3 Nanyang Technological University, Singapore, Singapore

**Keywords:** COVID-19, Rare disease, Rare disease organisation, Rare disease patients, Asia Pacific, Thematic analysis

## Abstract

**Background:**

This study assesses the areas and extent of impact of the Coronavirus Disease of 2019 (COVID-19) pandemic on rare disease (RD) organisations in the Asia Pacific region. There is no existing literature that focuses on such impact on RD organisations in any jurisdictions, nor RD populations across multiple jurisdictions in the Asia Pacific region. A cross-sectional survey was distributed to RD organisations between April and May 2020. Quantitative and qualitative data on the impact of COVID-19 on RD organisations and patients were collected from the organisation representative’s perspective. Qualitative data was analysed using thematic analysis. A follow-up focus group meeting was conducted in August 2020 to validate the survey findings and to discuss specific needs, support and recommendations for sustainable healthcare systems during the pandemic.

**Results:**

A total of 80 RD organisations from Australia, Hong Kong Special Administrative Region of China, India, Japan, mainland China, Malaysia, New Zealand, the Philippines, Singapore and Taiwan participated in the study. Of all, 89% were concerned about the impact of pandemic on their organisations. Results indicate that 63% of the organisations functioned at a reduced capacity and 42% stated a decrease in funding as their biggest challenge. Overall, 95% believed their patients were impacted, particularly in healthcare access, social lives, physical health, psychological health and financial impact. Specifically, 43% identified the reduced healthcare access as their top impact, followed by 26% about the impact on daily living and social life. Focus group meeting discussed differential impact across jurisdictions and point towards telemedicine and digitalisation as potential solutions.

**Conclusions:**

This serves as the first study to assess the impact of COVID-19 on RD patients and organisations across multiple jurisdictions in the Asia Pacific region, identifying major themes on the impact on both RD patients and organisations. By including 80 organisations from ten jurisdictions, our study presents the most comprehensive assessment of the pandemic’s impact to date. It highlights the need for mental health support and sheds light on moving towards telemedicine and digitalisation of organisation operation, which constitutes a sustainable model in times of pandemics and beyond.

**Supplementary Information:**

The online version contains supplementary material available at 10.1186/s13023-021-01766-9.

## Introduction

Since the outbreak of the Coronavirus Disease of 2019 (COVID-19) in Wuhan, China in December 2019, the pandemic had spread to many countries throughout the world. The outbreak was declared a public health emergency in January, officially categorised as a pandemic in March and had spread to more than 200 countries by April. During the period between 27 April and 23 May 2020, the total number of cases worldwide rose from 2,921,252 to 5,165,091 [1]. The COVID-19 pandemic has brought new challenges to all populations worldwide. Responsive measures at the national level largely prioritised infection control with overwhelmed health services focusing on treatment and management of those affected. The disastrous impact following the cessation of non-urgent healthcare services and transportation of medication was unprecedented for most but especially so for those with rare diseases (RDs).

According to the World Health Organisation (WHO), rare or orphan disease affects less than 5 per 10,000 people. It is believed that there are 5000–8000 RDs, affecting 400 million people, which is roughly 1 in 15 people (6.67%) worldwide [2]. Patients with RDs often experience life-long disability, life-threatening conditions or severely impacted quality of life. They require medical services to different extents, including rehabilitation, physiotherapy, regular hormone or enzyme therapy etc. The burden of these demands on physical, social, and psychological health has been severely exacerbated by a reduction in healthcare services and confinement measures on the marginalised RD populations within unequitable health systems. The situation has required patients and organisations to adapt and find alternative ways of support. RD patient organisations are typically registered, non-profit and patient-led communities that aim to promote awareness and advocate patient rights for RDs [3]. Different patient organisations cater to different specific RD conditions in a particular region. Organisations should be active and responsive to provide support and information to RD patients [4]. During the pandemic, patient organisations have been forced to expand their scope of services ranging from provision of personal protective gear, medication arrangement, to psycho-social/mental health support.

The assessment of such pandemic-related difficulties has attracted research interest across various geographies and health areas. However, research within the Asia Pacific region, accounting for over 60% of the global population, remains sparse [5]. Research on the impact of COVID-19 on the RD population is even more limited. To the best of our knowledge, there is no existing literature that has evaluated the impact of the pandemic on the functioning of RD organisations. Assessing the impact of COVID-19 on these organisations is critical in order to understand how the pandemic has impacted their functions and their patient groups, specific to geographic location and the type of RD. Further, we believe that patient organisations, working with various stakeholders such as healthcare professionals, patients, payers, health and non-governmental organisations, and industry partners, offer a unique perspective of the healthcare system. An assessment of their experiences allows us to draw on best practices and elicit insights into the wider health ecosystem during pandemic and beyond. This study, therefore, seeks to assess the impact of the pandemic on RD organisations and their members in the Asia Pacific region, analyse the major challenges that each stakeholder encountered and provide future directions for better preparedness in the future.

## Methodology

### Participants

A cross-sectional study was carried out from 27 April to 23 May 2020. The Asia Pacific Alliance of Rare Disease Organisations (APARDO), the regional alliance of RD patient organisations, developed a structured online survey to collect data from their members. Directors or the representatives of the RD organisations were recruited online. The information sheet and the survey, in English and Chinese, were distributed via the national RD federations to their member organisations across ten jurisdictions in the Asia Pacific region, including Australia, Hong Kong Special Administrative Region (HKSAR) of China, India, Japan, mainland China, Malaysia, New Zealand, the Philippines, Singapore and Taiwan. Participants were fully informed of the study objectives. Participation was completely voluntary, and completion and return of the survey implied consent.

### Study design and data analysis

The online survey contained ten quantitative and qualitative questions about the impact of the pandemic on individual organisation and their patients, the major challenges the organisation encountered, the changes brought about by the pandemic and special needs for the future. Both multiple-choice questions and open-ended questions were included. Majority of the questions were multiple-choice questions with one of the options allowing participants to further elaborate on their choice. These open-ended questions included the participant’s top three areas of concerns and impact of the pandemic on their patients and organisations, changes to organisational activities and patient support, biggest challenges or successes during the pandemic, and projects that could meet special demands of the organisation. Survey findings from multiple-choice questions were descriptively analysed. Open-ended questions were analysed using Braun & Clark’s framework for thematic analysis [6]. Analysis was reflexive, open coding was used and the qualitative responses were grouped into emerging semantic themes.

Data analysis was performed independently by two institutes—APARDO and the Clinical Genetics and Genomics Team, Department of Paediatrics and Adolescent Medicine, the University of Hong Kong. Initial discrepancies between the independent analyses were discussed and resolved, as consensus was reached after successive online meetings. Following multiple revision of the analysis, the two versions were merged.

Subgroup analysis by jurisdiction was also performed. Organisations from jurisdictions with three or fewer participants were regrouped based on three criteria: geographic proximity, similarities on healthcare systems, and socio-economic status. The five groups were (i) HKSAR China, (ii) mainland China, (iii) Australia and New Zealand, (iv) Japan, Singapore and Taiwan, and (v) India, Malaysia and the Philippines. Data from New Zealand was grouped with Australia for the proximity of geographic location as both are part of Oceania. Data from Japan, Singapore and Taiwan was grouped together as they are relatively socio-economically developed, with their national healthcare expenditure per capita all above USD1,000 in 2017. Support for patients, including healthcare system, governance, clinical expertise and funding are also relatively more established for the three jurisdictions [7]. In contrast, India, Malaysia and the Philippines were distinguished as a separate group because of their relatively lower socio-economic development, with the national health expenditure per capita below USD500 in 2017. In this group, access to healthcare for RD patients and funding for RD research and treatment are also currently under development [7]. Data from mainland China and HKSAR of China were analysed independently due to the relatively large number of participating organisations. Including these in any of the previous groups would have led to faulty generalisation given the lower engagement of patient organisations in the study.

### Focus group meeting

A follow-up focus group meeting was conducted online on 18 August 2020. The objective of the meeting was to validate results and interpretation, to reach consensus from participating organisations across the region, and to obtain additional perspectives that might have been ignored in the survey. All survey participants were invited to the online forum discussion. Other RD patients and organisation representatives who did not participate in the survey were also welcomed to attend the meeting. Preliminary results from data analysis were presented to the focus group participants. Open discussion was carried out and participants from RD organisations were invited to share their views, especially on the potential solutions and future directions during and beyond the pandemic. Finally, representative umbrella organisations were invited to continue to communicate with the project leaders with further thoughts. Some of the research findings from this current study have also been reported as a summary survey report shared by APARDO and Rare Diseases International on their organisation websites to facilitate knowledge exchange among the RD population.

## Results

### Participating rare disease organisations

A total of 80 RD organisations across ten jurisdictions participated in the survey during 27 April to 23 May 2020. Among the 80 RD organisations, ten are national RD federations and members of APARDO, while 70 are member organisations of the federations. The ten federations or alliances are responsible for the more comprehensive work for the RD populations, soliciting opinions of their member organisations and related stakeholders. The 70 member organisations have different target populations and roles, with some cater to specific RD conditions. The ten jurisdictions were Australia, HKSAR of China, India, Japan, mainland China, Malaysia, New Zealand, the Philippines, Singapore and Taiwan. Three organisations did not state their location (Table 1).

**Table 1:** Number of participating rare disease organisations in each jurisdiction

Jurisdictions	Number of participating organisations
Australia	15
HKSAR, China	18
India	2
Japan	2
Mainland China	30
Malaysia	3
New Zealand	1
Philippines	1
Singapore	2
Taiwan	3
Unknown	3
Total	80

*HKSAR* Hong Kong Special Administrative Region

For the follow-up focus group meeting, a total of 30 representatives participated in the forum discussion. All participants completed the survey that was conducted in April to May. Due to differences in time zones and other priorities, other organisations were not able to attend the meeting. Eight organisations returned post-meeting feedback to provide more detailed information on the COVID-19 situation in their jurisdiction, and the functioning and role of their organisation via email.

### Impact on rare disease organisations’ operation

Impact of the COVID-19 pandemic on RD organisations were classified into three themes using the thematic analysis approach: (i) organisations’ operation capacity, (ii) ease of supporting their members, and (iii) mode of communication.

#### Organisation’s operation capacity

Almost two-thirds (63%) of the organisations highlighted that they functioned at a reduced capacity or were completely non-functional during the study. Further, 24% functioned normally and 13% indicated increased activity. Of the total, 36 organisations further commented on the change in operation. More than half (55%) of the responses revealed that there was reduction in activities for both members and the organisation itself. Reduced organisational activities resulted from cancellation of events, forced closure of centres and insufficient manpower. In contrast, 18% of the responses raised that there were no changes in their organisations’ dynamics, because they had been utilising online platforms before the pandemic. On the other hand, 13% of the responses shared that the workload had increased as there were more requests from patients and new pandemic-related queries and tasks. In particular, staff had been busier coordinating with different organisations and distributing resources with patients. It was found that 42% of the organisations found increased volume of patient requests for help as one of their biggest challenges.

#### Supporting members

The impact on the continuity of patient support was also evaluated. Of all organisations, 63% considered support for members either more difficult or much more difficult, and 32% experienced no difference. Fifty-four organisations further shared the impact on patient support. The most compelling (49%) perception of the reason for such difficulties was the reduced physical interactions between organisation and members due to confinement measures that were exacerbated in cases of limited IT capacity. The reliance on online communication made supporting patients difficult as not all members were comfortable or competent with the technology. The second most frequently identified reason was the increased workload of the organisation. Of all, 22% of the responses described that due to staff shortage and increased pandemic-related matters, provision of effective support to patients was relatively difficult. Moreover, 20% reported reduced access to healthcare as a factor. This is because organisation representatives could not visit patients in hospital settings nor distribute medications to those living in more remote areas.

#### Communication

**Fig. 1:**
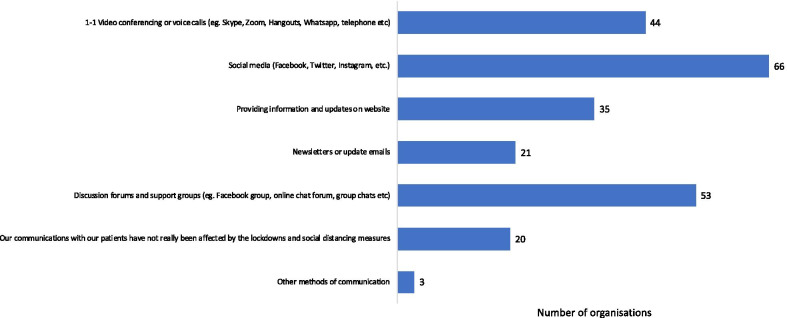
Mode of communication used by rare disease organisations (n = 79)

In terms of communication, 84% of the organisations used social media as one of the means to reach out to patients during lockdowns and social distancing. Among all participants, 67% made use of discussion forums and support groups to maintain support for members and 56% also used video conferencing and video calls (Fig. 1). Furthermore, “digitalisation of operation” was the most frequently identified reason for perception of organisational success during the pandemic. Of the 19 responses that shared their perceived successes, 52% mentioned that they had conducted meetings and consultations efficiently as well as shared information with members on a frequent basis via online platforms.

### Concerns from rare disease organisations

**Fig. 2:**
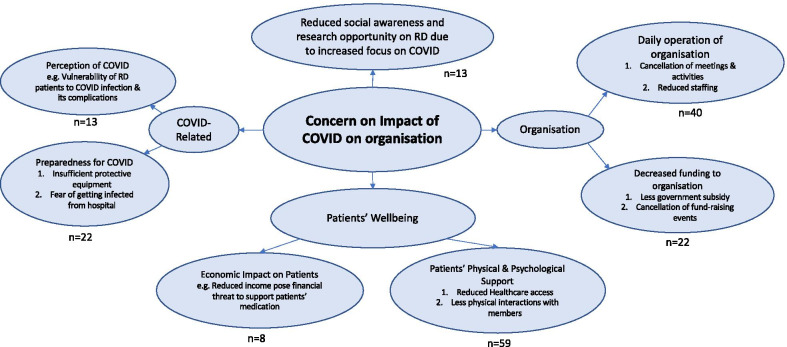
Thematic map on the top three areas of concern on the impact of COVID-19 on rare disease organisations

A total of 71 participants (89%) stated that they were concerned about the impact of COVID-19 pandemic on their organisations, in which 66% were very or extremely concerned. Sixty-seven participants further shared their top three areas of concern (Fig. 2). Four major themes on their concerns were identified: (i) patient’s well-being, (ii) organisation’s operation, (iii) perception of and preparedness for COVID-19, and (iv) awareness on RDs. The top three areas of concern from each jurisdiction-group were shown in Additional File 1.

#### Patient’s well-being

Majority of the organisations were concerned about their patients’ wellbeing and provision of physical and psychological support. It was found that 33% of the responses grouped under the theme “patients’ wellbeing” highlighted that  reduced healthcare access and physical interactions with members could impact the physical and emotional health of the beneficiaries. Besides physical and psychological impacts, concerns about economic impacts on patients were also raised, but with much smaller significance.

#### Organisation’s operation

The normal functioning of the organisations was also of great concern. Second to “patients’ wellbeing”, the theme “daily operation of organisation” was mentioned in 23% of the responses. This included cancellation of meetings and activities with members, as well as reduced staffing due to social lockdown measures. Concerns about decreased funding to organisations were identified. Among all responses, 42% of the organisations picked “decreased funding” as one of their biggest challenges, 21% included “sustaining financial support” as the key challenge during the pandemic and 12% indicated that fund-raising activities were cancelled and government subsidies were reduced. On the contrary, successes in maintaining the operation of the organisation were also reported.

#### Perception of and preparedness for COVID-19 & Awareness on rare diseases

Overall, 12% of the organisations mentioned about the concerns regarding the preparedness for the pandemic, for example having insufficient protective masks and personal protective equipment (PPE). Only 7% of all responses indicated the perception of COVID-19 as their top three concerns, which included worries about the vulnerability of RD patients to COVID-19 infection and its complications. Lastly, 7% responses mentioned how social awareness had shifted mainly to the pandemic such that opportunities for research and medical advancements in RD were halted.

### Impact on rare disease patients

Organisations believed that the top three difficulties encountered by their patients during the pandemic were access to medical care (66%), access to medications (58%) and access to PPE (55%).

**Fig. 3:**
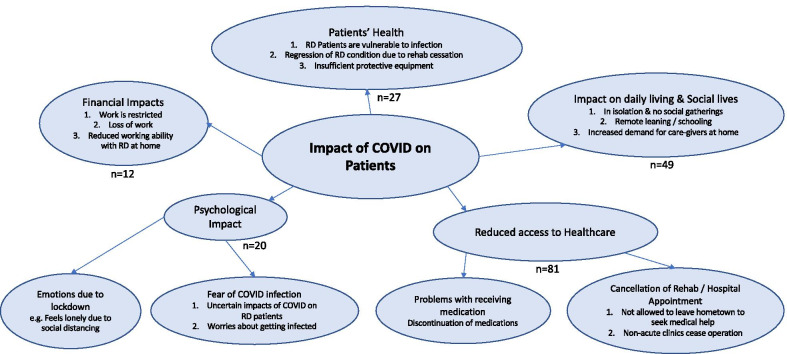
Thematic map on the top three areas of impact of COVID-19 on rare disease patients

Sixty-nine organisations shared the top three areas which they thought patients were most impacted on. Five most commonly identified themes were: (i) reduced healthcare access, (ii) social impact, (iii) physical health impact, (iv) psychological impact, and (v) financial impact (Fig. 3). The top three impacts identified from each jurisdiction-group were shown in Additional File 2.

The most frequently identified impact was “reduced healthcare access” (43%). Healthcare access, including hospital appointment, rehabilitation services (i.e. physiotherapy), non-acute clinics and distribution of medication, was affected due to the COVID-19 pandemic.

The second most frequently identified impact was “impact on daily living and social life” (26%). Participants described that “patients were mainly in isolation” and deprived of any social activities, and that “children had to be schooled via remote learning” during the pandemic.

Furthermore, 14% of the responses were related to physical health of patients. Participants believed that RD patients were more vulnerable to COVID-19 infection, and felt that they had insufficient protective equipment. Moreover, as a result of discontinuation of rehabilitation training, patients feared the regression of RD conditions.

Lastly, the impact on psychological health and financial status was a relatively smaller concern. Only 11% of the responses revealed that patients had to deal with emotions arising from social distancing and the anxiety brought about by the uncertainties of the virus. Sharing this concern, a respondent offered: “as a vulnerable segment of (the) society, being advised to self-isolate comes with significant mental health challenges”. Only 6% of the responses included financial impacts as the top three areas of impact on RD patients, indicated that loss of occupation and restricted working hours due to social lockdown posed threats to families’ finances.

### Subgroup analysis: By-jurisdiction group analysis

Table 2 shows the impact of COVID-19 pandemic on rare disease organisations and patients across different jurisdictions that participated in this study.

**Table 2:** Impact of COVID-19 on rare disease organisations and patients across the Asia Pacific region

	Overall: Asia Pacific	HKSAR China	Mainland China	Australia, New Zealand	Japan, Taiwan, Singapore	India, Malaysia, Philippines
**Impact on rare disease organisations’ operation**						
1. Daily operations	42%*	33%*	41%*	50%*	43%*	60%*
2. Reduced capacity or completely non-functional	63%	72%	48%	56%	86%	50%
3. Support patients’ physical & psychological wellbeing	55%*	47%*	59%*	63%*	43%*	40%*
4. Financial impacts (e.g. funding)	33%*	27%*	14%*	50%*	71%*	40%*
5. Support work made more difficult or much more difficult during the pandemic	63%	72%	47%	75%	71%	67%
6. Able to adapt and digitalise operations	58%*	0%*	63%*	100%*	50%*	0%*
7. Able to supply protective gear	21%*	50%*	13%*	0%*	50%*	0%*
**Impact on rare disease patients**						
1. Faced challenges accessing medical care or treatment / avoided seeking care for complications related to their rare condition	86%*	100%*	83%*	81%*	57%*	80%*
2a. Difficulty accessing medical care: scheduled appointments postponed or cancelled	68%*	67%*	79%*	63%*	57%*	60%*
2b. Had their rehabilitation therapies cancelled or postponed	12%*	20%*	8%*	6%*	0%*	20%*
3. Feeling concerned about their rare health condition because of COVID-19	20%*	13%*	17%*	31%*	14%*	40%*
4. Difficulty accessing medicines	58%	56%	73%	56%	14%	50%
5. Trouble procuring personal protective equipment	55%	78%	50%	56%	71%	17%
6. General impacts [% of patients impacted in some degree; % of patients that were very or extremely impacted]	95%; 57%	89%; 50%	93%; 45%	100%; 69%	100%; 43%	100%; 83%
7. Negative impact on physical health or wellbeing	36%*	40%*	33%*	38%*	29%*	40%*
8. Negative impact on mental health or well-being	29%*	27%*	13%*	63%*	0%*	60%*
9. Negative impact on social health or well-being	57%*	80%*	38%*	56%*	57%*	80%*
10. Negative financial impact (i.e. loss of income or job)	14%*	0%*	17%*	25%*	29%*	0%*

*Based on top 3 areas provided as qualitative responses

*COVID-19* Coronavirus Disease of 2019, *HKSAR* Hong Kong Special Administrative Region

Findings highlight a considerable diversity among organisational responses across different jurisdictions. Firstly, digitalisation of operation was a success in Australia and New Zealand as all organisations from these two jurisdictions were able to adapt and digitalise their operations. Other jurisdictions were not able to adapt as fast. This might be due to the lack of technological platforms for organisations to digitalise their operations. Second, organisations in mainland China were relatively less affected in terms of daily operation of the organisation. Only less than half (48%) of the organisations in mainland China functioned at a reduced capacity. Functioning of the organisations in Japan, Taiwan and Singapore were most affected, with 86% functioned in a reduced capacity. The reasons behind the discrepancies include organisations’ previous experience in online operation and the differential impact of COVID-19 during the study period. Third, RD patients in Japan, Taiwan and Singapore faced relatively smaller difficulties when accessing healthcare. Whilst all other jurisdictions had over 80% of patients mentioned challenges in accessing medical care or treatment, only 57% in Japan, Taiwan and Singapore stated so. In addition, none of the respondents from Japan, Taiwan and Singapore stated that their rehabilitation therapies had been cancelled or postponed. Lastly, concerns regarding mental health or well-being of patients varied substantially across the region. In particular, 63% of the organisations in Australia and New Zealand and 60% of the organisations in India, Malaysia, and the Philippines identified negative psychological impact on their patients as a result of COVID-19, while none in Japan, Taiwan and Singapore identified this as their top three impacts on their patients. However, when participants were asked about the type of support they wish to receive in the future, “mental health for patients” were the most chosen option (51%).

#### Support for rare disease populations

Participants chose the type of assistance they would want to receive regarding their needs during the pandemic. Majority (51%) picked “mental health support for patients”, followed by “funding for special projects” (47%), “advocacy training” (45%) and “connecting organisations and patients with reliable sources of information & expertise” (45%).

## Discussion

An analysis of the results highlighted serious challenges encountered by RD organisations and patients. Notwithstanding, pandemic-related research focus has targeted treatment regime for COVID-19 and its epidemiology, and largely overlooked its impact on RD patients and organisations who have been severely affected [8]. For patients, the discontinuation of hospital services and rehabilitation, supply of medication and psychological impact of social isolation have been significant. Similarly, patient organisations have found it demanding to change their mode of operation and digitalise patient support activities.

This study demonstrated the challenges that the organisations faced during the COVID-19 pandemic. Three major areas of impact were identified, being the organisations’ operation capacity, ease of supporting their members, and the change in mode of communication. During the focus group meeting where participants further shared their views on the research findings, one of the major reasons for the organisational challenges was due to financial hardships. Fundraising events have been cancelled, and organisations are receiving less government subsidies. This was in line with the research findings from the PatientView 2020 survey, in which 67% of the 1,720 respondent patient groups reported a decrease in their revenue during 2020 [9, 10]. In order to overcome disruptions to their functioning, RD patient organisations are expecting an accelerating shift to digitalising organisation’s operation.

The importance of digitalisation for all but especially RD populations has been underscored by findings of this study. This study demonstrated that organisations with greater digital capacities had a lower negative impact on operations. Since the start of COVID-19 outbreak, physical workplace has become a danger for infection transmission when virtual space rose with popularity [11]. Converting face-to-face businesses into online meetings is now a global trend and need. Organisations in the Asia Pacific region that claimed their operation were not affected or were less impacted by the pandemic were those who already had their operation digitalised. For example, all organisations in Australia and New Zealand were able to adapt and digitalise their operations, and were less severely affected. Organisations “felt the need to move more of their services online” and reflected that “movement restrictions policies has taught us quickly to operate a lot more virtually and effectively to the extent that we decided we no longer need a permanent office… which also saves overhead costs”. The adaptability of organisations in managing to pivot to the changing landscapes and digitalise their operations and increase the sharing of information and resources with partner organisations indicate their resilience and creativity. It also constitutes an alternate and sustainable model of health for patient population in the region. Digitalisation of operation is likely to be the new normal.

Patient organisations have become a major lifeline for RD patients living in the community. The impact on RD organisations has further exacerbated the difficulties and challenges faced by their patients. The impact of the pandemic has been previously reported on RD patients in HKSAR China, Ireland, the United States of America (USA), Canada, and Europe [8, 12–15]. Evidence in the Asia Pacific region was limited to the study in HKSAR China [8]. The four studies [12–15]. beyond the Asia Pacific region were reviewed to compare the pandemic’s impact on RD populations in other regions and in the Asia Pacific region (Table 3). Severe heterogeneity in participant characteristics and study methodology precluded statistical comparisons. Nevertheless, this gives an overview of the pandemic’s impact on RD patients in different parts of the world. Impact on organisations could not be compared as the current study remains to be the only evidence available in literature.

**Table 3:** Impact of COVID-19 on rare disease patients in different regions across the world

	Europe [12]	USA [13]	Canada [14]	Ireland [15]	This study: Asia Pacific region
1. Faced challenges accessing medical care or treatment	48%	32%	Nearly 50%	31%	66%
2. Difficulty accessing medical care: scheduled appointments postponed or cancelled	66%	79%	Nearly 50%	53%	68%*
3. Feeling concerned about their rare health condition because of COVID-19	/	/	75%	73%	20%*
4. Difficulty accessing medicines	21%	14%	Nearly 40%	/	58%
5. Trouble procuring personal protective equipment	/	9.5%	/	/	55%
6. General impacts [% of patients impacted in some degree; % of patients that were very or extremely impacted]	/	92%	/	/	95%; 57%
7. Negative impact on physical health or wellbeing	59%	/	/	59%	36%*
8. Negative impact on mental health or well-being	67%	94%	Nearly 50%	62%	29%*
9. Negative impact on social health or well-being	/	/	/	/	57%*
10. Negative financial impact (i.e. loss of income or job)	/	37%	/	33%	14%*

*Based on top 3 areas provided as qualitative responses

All of the studies were conducted during April–June 2020

*COVID-19* Coronavirus Disease of 2019, *USA* United States of America

A major disparity across the jurisdictions was mental health issues. Impact on mental health was less of a concern in the Asia Pacific region as compared to Europe, the USA, and Canada. Compared to the 94% in the USA [13] and 67% in Europe [12], only 29% of the responses in this study mentioned mental health as one of their top three concerns. While this might suggest that the mental health of patients in Asia Pacific region are less impacted, it might also indicate that populations in the region are less aware or attentive of their psychological needs. The discrepancies across different regions could also be due to cultural differences, perspectives and stigma about mental health issues in different jurisdictions [16]. Previous studies have suggested that Asians are less willing to discuss their psychological states or moods because of fears of social stigma and shame [17]. The ability to internalise the stress and emotions for the sake of community propriety is regarded to be extremely important in most Asian populations [16]. Keeping this mind, the provision of sufficient resources for organisations to support the mental health of patients and their carers experiencing months of isolation in the Asia Pacific region is equally critical.

COVID-19 has significantly accelerated digitalisation in all sectors across the globe for continuation of services traditionally delivered face-to-face. One such critical service has been telehealth. Telehealth has been widely in use in other regions of the world and can be seen as an effective tool to replace face-to-face medical care. Telehealth includes all activities that deliver care without direct physical contact. It can be conducted synchronously via telephone and video, or asynchronously through online portals and virtual agents [18]. Approximately 83%, 52%, and 31% of patients in the United States, Europe, and Ireland respectively were offered or had medical appointments remotely [12, 13, 15]. Strongly supported by clinicians and the general public, telehealth has been seen as a convenient and infection risk-free platform to improve healthcare accessibility [19]. In contrast, medical appointments conducted using telehealth were less reported in the Asia Pacific region, where 23% of the organisations mentioned difficulty in accessing telehealth. During the follow-up focus group discussion forum, a major theme brought up by the representatives was the digitalisation of medical services. Table 4 listed out the quotes from the organisation representatives on digitalisation of health and operation. Patients highlighted this as the best possible alternative for sustaining delivery of medical care in the midst of confinement measures. “One major benefit is that we have moved to a new mode of healthcare—telehealth, which is the future”, a representative from a RD organisation shared in the focus group meeting. Advantages of telehealth also include balancing the supply of clinical services across geographical boundaries, providing mental support to isolated patients and conservation of personal protective equipment for frontline workers [18].

**Table 4:** Quotes from rare disease organisation representatives from focus group meeting and post-meeting feedback: digitalisation of health and operation

Jurisdiction	Quotes on telehealth/digitalisation of operation
Australia	“Many chose to restrict using health and other public services. However, many health services have been rapidly shifted to use telemedicine which certainly helps.”
HKSAR China	“The organisation feels the need to move more of their services online. More webinars and annual general meetings are moving online. Some NGOs are pushing rehabilitation programmes for the elderly online, but the organisation is still working on that for Rare Disease patients.”
Japan	“The number of patients who receive regular medical examinations for chronic diseases has been extended to include online medical services through telephone calls to doctors and other means, so that patients can receive their prescriptions at the pharmacy of their choice without having to visit a hospital.”
Malaysia	“We launched our tele-physio programme, everything was pulled together and run virtually. It’s successful and moving to Phase II. What's interesting is that the movement restrictions etc. has taught us quickly to operate a lot more virtually and effectively, to the extent that we decided we no longer need a permanent office and in the process of shifting to flexible shared office and storage, and home-office arrangements, which also saves overhead costs.” “All stakeholders (are) a lot more open and willing, and (are) more available and timely, to be on digital channels.”
New Zealand	“Due to COVID, a major benefit is that we have moved to a new mode of healthcare- telehealth, which is the future. Originally, there are lots of barriers to telehealth, such as government regulations and insurance. However, due to COVID, the transition to telemedicine has accelerated, which is beneficial to rare disease patients. As such, this COVID has forced us to move with legal and insurance issues surrounding telehealth. This is also true for allied health services such as physiotherapy or mental health.”

*COVID* coronavirus disease, *HKSAR* Hong Kong Special Administrative Region, *NGOs* non-governmental organisations

This study demonstrated differential impact of the COVID-19 pandemic on RD organisations and patients across jurisdictions in the Asia Pacific region. The disparate consequences on RD organisations might be due to varied organisational sizes and functions. Reduced functional capacity of organisations of similar size in the same jurisdictions might also result in different outcomes due to their different roles. While organisations that focus more on the mental support of patients might be less impacted due to the usage of online platforms, other organisations responsible for the supply of medications were severely impacted due to disruption of supply chains during the pandemic. The differential impact of COVID-19 pandemic on RD organisations and patients in different jurisdictions could also be explained by the healthcare policies and measures that the governments adopted across the region. Mainland China was the first jurisdiction to be impacted during the outbreak of COVID-19. The mainland Chinese government imposed strict lockdown measures and large-scale quarantine that led to massive reduction of infection in the second wave [20]. This might explain why RD organisations in mainland China were relatively less impacted in terms of functioning as this survey was carried out at a later stage of the pandemic in mainland China. Patient groups had varied views on their home government’s ability to manage the pandemic, with mainland China considering their government to be doing a “very effective” job at tackling the pandemic [9, 10]. In HKSAR China, Singapore and Taiwan, governments and citizens used their experience from the 2003 SARS outbreak and were more prepared to respond proactively and quickly [21]. In contrast, in low/ middle-income countries, the healthcare systems were likely underequipped and less prepared for the pandemic [22]. Governments’ adoption and policy of telemedicine also played a role on the differential impact. Even before the pandemic, Australia, mainland China, and Singapore were recognised as early adopters of telemedicine with supporting policies, strong government support and cultural receptance. On the contrary, Japan and HKSAR were only at a pilot stage with no regulatory guidance and leading companies as pioneers [23]. Early adopters had laid notable groundwork which facilitated the growth of telemedicine during the pandemic, thus faced less difficulty in accessing healthcare.

While negative impact of the pandemic has been perceived, it has also brought opportunities for solutions and for RD communities and stakeholders to collaborate and support each other during the COVID-19 crisis. In fact, 29% of the organisations in the current study identified “forming connection with other RD organisations and external support” as their success during the pandemic. Patient organisations were the first port of call in the pandemic, and they had strengthened their position and reinforced their role in the RD community [10]. This study indicates the criticality for policy makers to recognise patient vulnerabilities and the pivotal role of RD organisations during the COVID-19 pandemic. The RD populations must be protected and given special attention and consideration when policies are designed. In particular, previous study illustrated a marginalisation and oversight of RD patient needs and consideration in response to the pandemic [24]. Meanwhile, policy makers should ensure fair allocation of resources including the distribution of protective equipment, being aware of the impact on RD patients after diverting majority of the healthcare resources to fight the pandemic. In order to protect the already vulnerable patient groups, health officials and leaders could learn from this pandemic to become more diverse and tailor appropriate responses for the future [18]. In the long run, telehealth is undeniably the way forward. However, governments must be on guard of the potential legal and ethical issues. Preparation and surveillance with legislation should be performed in order to gain trust from the public. Policy makers would have to monitor the development of the new digital era.

This study also acknowledges several limitations. First, participants were self-selected on a strictly voluntary basis. RD organisations with limited access to internet or online technology might not be able to participate in this study. Second, the number of organisations were imbalanced between jurisdictions. Jurisdictions with a small number of participating organisations limited generalisability to other organisations in the same jurisdiction. RD organisations’ function and size might differ and thus affecting the impact of COVID-19 on individual organisation. Third, findings and conclusions were based on both quantitative and qualitative responses. However, like many of the qualitative studies, missing data in qualitative responses were relatively common. In addition, since participants were only asked to share at most three aspects in each qualitative question, other unmentioned aspects could not be identified. Impact on the RD patients and organisations might have been underestimated. Fourth, since the survey was conducted from April to May, the findings only represent the impact of COVID-19 during that period, in which the pandemic affected differentially in each jurisdiction. Last, survey responses and focus group sharing were based on the representatives or RD organisations, direct comparison with other studies is difficult. However, this is arguably the most efficient and effective way to reach out to research in RD populations especially during a pandemic, as RD patients are managed by different specialties, and many have even delayed or cancelled their appointments during this time, making recruitment more difficult.

While this cross-national survey study has its limitations, this provides empirical evidence that warrants policy makers to plan ahead to facilitate enhanced preparedness levels for the possible resurgence of COVID-19 infection in the future. Recognising the differential impact of the pandemic on the RD population, this study suggests the importance of including patient representatives in response preparedness. It is recommended to provide urgent mental health support for RD patients, funding of special projects that supports resilience and preparedness, advocacy training for improved capacity within patient organisations, and facilitate access to reliable sources of information and expertise among RD organisations. It argues a greater investment in patient organisation capacity building for sustaining their critical role in meeting the needs of the under-served RD populations in the Asia Pacific and beyond.

## Conclusion

This study illustrates the impact of COVID-19 on RD patients and organisations across the Asia Pacific region. It acts as a statement to urge leaders of the Asia Pacific region to protect the physical and psychological vulnerability of RD populations and evaluate current lockdown policies to ensure adequate healthcare access and medication supply. This study does not only highlight the need for mental health support to patients and caregivers, it also indicates the value of digitising operation of RD organisations, and sheds light on the possibility of integrating telemedicine across all health services. These measures require strengthening of health systems and innovative approaches for enhanced capacity, which constitutes an alternate and sustainable model in times of and beyond pandemics.

## Supplementary Information

**Additional file 1**. Top three areas of concern on organisations from each jurisdiction group. A colour-coded table showing different areas of concern on the impact of COVID-19 on rare disease organisations from each jurisdiction group

**Additional file 2**. Top three areas of impact on patients from each jurisdiction group. A colour-coded table showing different areas of impact of COVID-19 on rare disease patients from each jurisdiction group

## Data Availability

The data used and/or analysed during the current study are available from the authors upon reasonable request.
