# Supplementary material for: A thematic study: impact of COVID-19 pandemic on rare disease organisations and patients across ten jurisdictions in the Asia Pacific region
Source: Orphanet J Rare Dis. 2021 Mar 5;16:119. doi: 10.1186/s13023-021-01766-9 (PMC7935006; doi:10.1186/s13023-021-01766-9)
Supplement: Supplementary file 1 — Additional file 1. Top three areas of concern on organisations from each jurisdiction group. A colour-coded table showing different areas of concern on the impact of COVID-19 on rare disease organisations from each jurisdiction group [file 13023_2021_1766_MOESM1_ESM.pdf]

**Additional File 1.** Top three areas of concern on the impact of COVID-19 on organisations from each jurisdiction group

<table>
<tr><th></th><th>Most frequently identified</th><th>2nd most frequently identified</th><th>3rd most frequently identified</th><th>Least frequently identified</th></tr>
<tr><td rowspan="2">Overall (n=177)*</td><td rowspan="2">Patients’ physical and psychological support (n=59; 33%)</td><td rowspan="2">Daily operation of organisation (n=40; 23%)</td><td>Preparedness for COVID-19 (n=22; 12%)</td><td rowspan="2">Economic impact on patients (n=8; 5%)</td></tr>
<tr><td>Decreased funding to organisation (n=22; 12%)</td></tr>
<tr><td rowspan="2">HKSAR, China (n=41)*</td><td>Patients’ physical and psychological support (n=12; 29%)</td><td rowspan="2">Daily operation of organisation (n=7; 17%)</td><td>Decreased funding to organisation (n=4; 10%)</td><td>Economic impact on patients (n=1; 2%)</td></tr>
<tr><td>Preparedness for COVID-19 (n=12; 29%)</td><td>Perception of COVID-19 (n=4; 10%)</td><td>Reduced awareness on rare diseases (n=1; 2%)</td></tr>
<tr><td>Mainland China (n=55)*</td><td>Patients’ physical and psychological support (n=23; 42%)</td><td>Daily operation of organisation (n=14; 25%)</td><td>Economic impact on patients (n=5; 9%)</td><td>Perception of COVID-19 (n=2, 4%)</td></tr>
<tr><td>Australia, New Zealand (n=43)*</td><td>Patients’ physical and psychological support (n=13; 30%)</td><td>Daily operation of organisation (n=11; 26%)</td><td>Decreased funding to organisation (n=8; 19%)</td><td>Preparedness for COVID-19 (n=0; 0%)</td></tr>
<tr><td rowspan="3">Japan, Singapore, Taiwan (n=18)*</td><td rowspan="3">Decreased funding to organisation (n=5; 28%)</td><td>Patients’ physical and psychological support (n=4; 22%)</td><td rowspan="3">Reduced awareness on rare diseases (n=1; 6%)</td><td rowspan="2">Economic impact on patients (n=0; 0%)</td></tr>
<tr><td>Daily operation of organisation (n=4; 22%)</td></tr>
<tr><td>Preparedness for COVID-19 (n=4; 22%)</td><td>Perception of COVID-19 (n=0; 0%)</td></tr>
<tr><td rowspan="3">India, Malaysia, Philippines (n=14)*</td><td rowspan="3">Daily operation of organisation (n=4; 29%)</td><td rowspan="3">Perception of COVID-19 (n=3; 21%)</td><td>Patients’ physical and psychological support (n=2; 14%)</td><td rowspan="3">Economic impact on patients (n=0; 0%)</td></tr>
<tr><td>Decreased funding to organisation (n=2; 14%)</td></tr>
<tr><td>Preparedness for COVID-19 (n=2; 14%)</td></tr>
</table>

Legend: Impact on organisation | Patient’s well-being | COVID-related aspect | Awareness on rare diseases

* Participants could list up to 3 areas of concern

**COVID-19** coronavirus disease of 2019; **HKSAR** Hong Kong Special Administrative Region
