# Supplementary material for: A thematic study: impact of COVID-19 pandemic on rare disease organisations and patients across ten jurisdictions in the Asia Pacific region
Source: Orphanet J Rare Dis. 2021 Mar 5;16:119. doi: 10.1186/s13023-021-01766-9 (PMC7935006; doi:10.1186/s13023-021-01766-9)
Supplement: Supplementary file 2 — Additional file 2. Top three areas of impact on patients from each jurisdiction group. A colour-coded table showing different areas of impact of COVID-19 on rare disease patients from each jurisdiction group [file 13023_2021_1766_MOESM2_ESM.pdf]

Additional File 2. Top three areas of impact of COVID-19 on rare disease patients from each jurisdiction group

	Most frequently identified	2nd most frequently identified	3rd most frequently identified	Least frequently identified
**Overall (n=189)***	Reduced access to healthcare (n=81; 43%)	Patient's daily living and social life (n=49; 26%)	Patient's physical health (n=27; 14%)	Financial impact (n=12; 6%)
**HKSAR, China (n=42)***	Reduced access to healthcare (n=19; 45%)	Patient's daily living and social life (n=13; 31%)	Patient's physical health (n=6; 14%)	Financial impact (n=0; 0%)
**Mainland China (n=65)***	Reduced access to healthcare (n=32; 49%)	Patient's daily living and social life (n=14; 22%)	Patient's physical health (n=10; 15%)	Patient's psychological health (n=3; 5%)
**Australia, New Zealand (n=49)***	Reduced access to healthcare (n=17; 49%)	Patient's psychological health (n=12; 24%)	Patient's daily living and social life (n=9; 18%)	Financial impact (n=4; 8%)
**Japan, Singapore, Taiwan (n=14)***	Patient's daily living and social life (n=6; 43%)	Reduced access to healthcare (n=4; 29%)	Financial impact (n=2; 14%)<br>Patient's physical health (n=2; 14%)	Patient's psychological health (n=0; 0%)
**India, Malaysia, Philippines (n=16)***	Reduced access to healthcare (n=5; 31%)<br>Patient's daily living and social life (n=5; 31%)	Patient's psychological health (n=4; 25%)	Patient's physical health (n=2; 13%)	Financial impact (n=0; 0%)

Reduced healthcare access	Physical health impact	Social impact	Psychological impact	Financial impact

* Participants could list up to 3 areas of patient impact

COVID-19 coronavirus disease of 2019; **HKSAR** Hong Kong Special Administrative Region
